# Diagnostic challenges and updated therapeutic strategies of Kimura’s disease: A case report successfully treated by dupilumab and review

**DOI:** 10.1097/MD.0000000000034191

**Published:** 2023-11-24

**Authors:** Stephanie Cordeil, Olivier Hermine, Arnaud Hot

**Affiliations:** a Department of Hematology, Lyon Sud Hospital, Hospices Civils de Lyon, Pierre-Bénite, France; b Claude Bernard Lyon 1 University, Lyon, France; c Department of Hematology and Imagine Institute, INSERM UMR 1163 and CNRS ERL 8254, Laboratory of Cellular and Molecular Mechanisms of Hematological Disorders and Therapeutic Implication, Necker Hospital, Paris, France; d University of Paris Descartes, Paris, France; e Department of Internal Medicine, Edouard Herriot Hospital, Hospices Civils de Lyon, Lyon, France.

**Keywords:** case report, corticosteroids, dupilumab, eosinophilia, Kimura’s disease, relapse

## Abstract

**Rationale:**

Kimura's disease (KD) is a rare and chronic eosinophilic related-disease, characterized by subcutaneous tissue masses, regional enlarged lymph nodes, hypereosinophilia and elevated serum IgE. KD usually affects young adults in the Asian population. In Western countries, the clinical and biological presentation of KD is often unknown, delaying the diagnosis. Therapeutic management is not standardized and despite recent advances, remission from KD can be difficult to achieve, especially in relapse situations.

**Patient concerns:**

We report the case of an non-Asian man with KD, initially misdiagnosed as lymphoma. We focus on his long-lasting clinical course with 20 years of recurrence despite several therapeutic lines.

**Diagnoses and interventions:**

We have emphasized the key points of the KD diagnostic challenge. We chose to focus on hemopathies as diagnostic traps to illustrate several overlapping features that blur frontiers with KD. With regard to treatments, lessons can be learned from the use of the therapeutic backbone, which relies on excision surgery, radiotherapy and corticosteroids.

**Outcomes:**

Advancements in KD pathogenesis have highlighted the pivotal role of Th2 lymphocytes driving eosinophil activation. Directly inspired by eosinophilic and allergic field practices, targeted therapies, such as dupilumab, provide hope for potential curative options.

**Lessons:**

Finally, we propose a therapeutic plan to treat newly diagnosed KD and discuss options for relapsing entities.

## 1. Introduction

Kimura’s disease (KD), also named “eosinophilic hyperplastic lymphogranuloma,” is a rare entity belonging to the spectrum disorder of chronic hypereosinophilia (HE).^[1]^ Sustained elevated eosinophilic blood levels are hallmarks of KD. Under unknown conditions, activated eosinophils secreting eosinophil-derived products, migrate into tissues and participate in local inflammation and damages.^[2]^

The first description of KD was published in 1937 by Kim and Szeto, whereas its first individualization as a separate disease was established in 1948 by Kimura et al. Typical clinical presentation first encompasses subcutaneous masses that are solitary or sometimes multiple, asymmetric, painless and mainly present in the head and neck region with a predilection for the post-auricular zone. Other localizations, such as the extremities of the upper limbs, oral cavity, orbital region and epiglottis have been reported. The average size has a diameter of 5 cm but masses can reach 20 cm. Overlying skin usually lacks particularities, nevertheless, pruritus and melanin pigmentation can be observed in 20% to 30% of patients. Another part of the clinical presentation is cervical lymphadenopathy of the tumor drainage area, which is documented in more than half of the cases. Finally, the swelling of the salivary glands, mostly the parotid glands, is observed. General signs such as asthenia, weight loss and night sweets, are often absent.

With regard to the targeted organs, KD can be considered a localized and benign HE-related disorder. Indeed, life-threatening organ involvement remains very uncommon in KD patients. To our knowledge, only 2 severe cases are reported in the literature with respectively an acute myocarditis and a Loeffler’s fibroblastic endocarditis.^[3,4]^ Association with ulcerative colitis is also rare.^[5,6]^ Renal dysfunction characterized by a nephrotic syndrome with several described types of glomerular histopathologies, is the most frequent systemic complication, occurring in 10% to 20% of patients.^[7,8]^

In addition to constant peripheral eosinophilia, blood analyses classically mention elevated IgE concentrations. C-reactive protein levels are usually normal or mildly increased.

Histopathological patterns flesh out the diagnosis. The lymph node architecture is still conserved. Massive follicular hyperplasia is observed in reactive germinal centers with respect to the mantle zone. Many eosinophils infiltrate the germinal centers, extrafollicular areas, lymph node sinuses, and extranodal tissues. The proliferation of postcapillary venules is described. The previous features are invariable in KD, whereas the following ones are less robust in KD histology. Variable perivascular fibrosis, mast cells infiltration in parafollicular areas, eosinophilic micro-abscesses, or IgE deposition in the germinal center are occasionally present.^[9]^ Compared to lymph nodes, skin and salivary gland specimens display a similar inflammatory pattern, with notably ectopic lymphoid structures.

Overall, the disease course is favorable with an excellent prognosis. Onset is quite insidious, lesions grow slowly, and frequently become chronic. Many patients present with numerous inflammatory flares triggered by unidentified factors that resolve spontaneously or with treatments.

Concerning its epidemiology, KD preferentially affects the young Asian population between 30 and 40 years of age. The male/female ratio is unbalanced at approximately 6:1. Potential genetic backgrounds and/or environmental stimuli required for KD pathogenesis, could explain this particular ethnicity distribution. Cases are rarely described in non-Asians or in patients of any age group, from pediatrics to geriatrics. When it occurs in Western populations, KD has a similar clinicopathologic presentation.^[10]^

**Figure 1.**
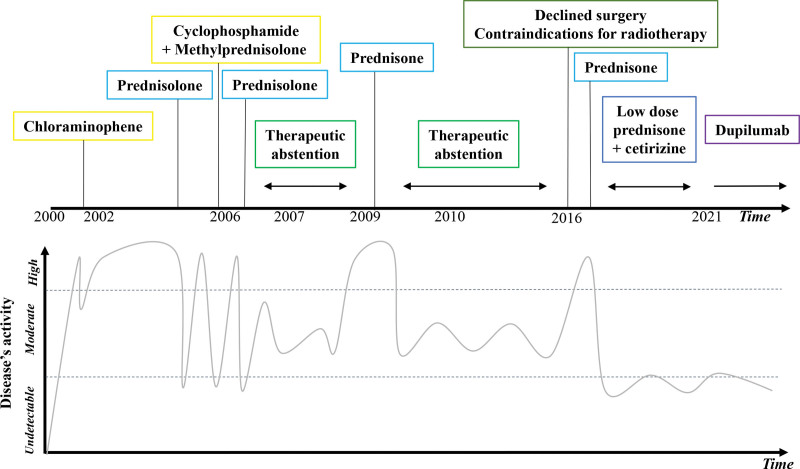
Schematic illustration of the patient clinical course according to the therapeutic choices. Disease’s activity is modelized on size estimation of the main orbital tumor.

Since its first description in 1937, and despite numerous published case reports over the last 20 years, KD is currently a diagnostic and therapeutic challenge, as illustrated in our patient’s prolonged history. Based on his medical management for 20 years, from the 2000s up to the present day (Fig. 1), we discuss the state-of-the-art of the KD diagnostic process, lymphoproliferative disorders as differential diagnoses not to be missed, and therapeutic modalities.

## 2. Clinical case

We present the case of an Algerian native 77-year-old man with a history starting at age 55 by the discovery of a fluctuating frontal mass. He had no significant past personal and familial medical history. He did not take any drugs and denied atopic manifestations.

Physical examination revealed swelling of the left sub-orbital region, extending to the temporal zone. The lesion was painless and nonerythematous. The patient underwent a first biopsy, which revealed a nonspecific inflammatory reaction of the cutaneous tissues.

**Figure 2.**
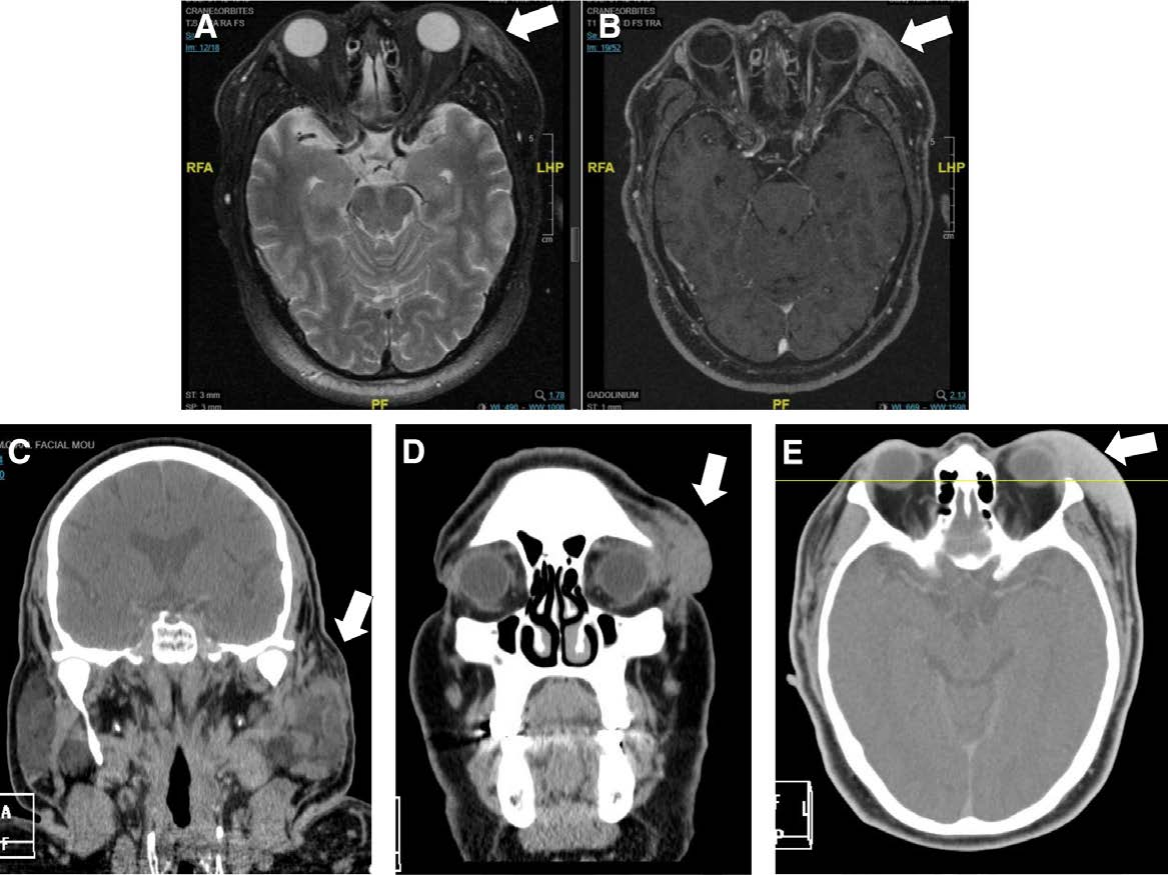
Radiological aspect of KD lesions on MRI and CT scan. Axial MRI: swelling in the upper left eyelid level, appearing as discrete T2 hypersignal (A) and enhanced after injection of gadolinium (B). Deep invasion or bone lysis was not observed. Coronal (C, D) and axial (E) CT scans. Hypertrophy of both parotid glands and within the left one, presence of an infiltrating tissue lesion, and partitioning in places (C). Lesion of the left latero-orbital soft tissue, coming into contact with the eyeball (D), was enhanced after injection (E). CT = computed tomography, KD = Kimura's disease, MRI = magnetic resonance imaging.

Because of persistent eyelid lesion, newly associated with ipsilateral parotid swelling and subangulomaxillary lymphadenopathies, physicians have raised the hypothesis of a lymphoproliferative syndrome. No constitutive symptoms (fever, weight loss, or night sweats) or local compressive complications were recorded. In 2002, a second more complete biopsy examined by experts in the lymphoma field made the diagnosis of an histologically atypical non-Hodgkin’s lymphoma mucosa-associated lymphoid tissue type. Chest and abdominal computed tomography scan, bone marrow biopsy and gastroscopy found limited head and neck disease. The patient started oral chemotherapy based on chloraminophene. Clinicians were very surprised by the orbital swelling progression during therapy. At the time, biological tests revealed chronic eosinophilia. Magnetic resonance imaging (MRI) pointed the rise of contrast-enhanced infiltrative masses into orbital and parotid tissues (Fig. 2). This unexpected evolution under chloraminophene, as well as the uncommon presentation, led to perform a repeated biopsy, mainly to eliminate a transformation toward a high-grade lymphoma. Histological analysis showed a globally conserved nodal architecture. Lymphoid hyperplasia was observed, but there was no evidence of clonal immunoglobulin heavy chain or T-cell receptor by polymerase chain reaction. Intense eosinophil infiltrates were identified. In addition, serum IgE concentrations were 1413 kU/L (N < 150 kU/L). Taken together with the benign disease course, a diagnosis of KD was finally suggested after 3 years of management.

The patient was then lost to follow-up for 1 year before visiting the physician for important orbital and parotid swellings with multiple supracentimetric cervical lymphadenopathies. Hence, the parotid nodules were rapidly analyzed using fine-needle aspiration cytology, which demonstrated no malignant cells. Thus, the diagnosis of a KD flare was comforted, sparing another biopsy. Treatment with intravenous prednisolone for 2 months resulted in complete regression of the tumors, which lasted 7 months. Subsequently, a heavy exacerbation occurred with a simultaneous unforeseen enlarged supraclavicular lymph node, which once again questioned the hypothetical malignant evolution of KD. Superficial parotidectomy was ordered, and the analysis concluded to KD. To prolong the glucocorticoid effect, methylprednisolone 0.5 mg/kg/d *per os* was associated with cyclophosphamide (for its immunosuppressive properties) 150 mg/d *per os* for 1 week, every month. This association quickly succeeded in regressing tumor size. However, 7 months after the completion of 6 courses, painless orbital tumefaction was detected again. During 2 years of therapeutic abstention, the patient experienced intermittent exacerbation of persistent masses with significant edema that lasted 15 days to 1 month. It was decided not to return to previous treatment because of its leukemogenic power.

In 2010, therapeutic options were discussed in a multidisciplinary meeting. Surgery appeared to be feasible according to the ophthalmologist, but the patient refused. Local radiotherapy was rejected because of potential long-term complications, such as secondary carcinogenesis and risk of cataract. Given the obese metabolic profile of the patient, who developed sleep apnea (without relation to KD) and diabetes, a “watch and wait” strategy was collectively chosen.

Tumefactions remained broadly stable until 2016, when they started to become larger, painful, and very itchy. Prednisone 0.5 mg/kg/d and cetirizine 10 mg/d lowered pruritus, inflammation and pain. Tapering corticosteroid led to fast relapse of edema and pruritis. Without any efficient alternative, it was decided to pursue a maintenance dose of oral steroids between 5 and 10 mg/d, increased to 25 to 50 mg/d in the acute phases.

In 2021, after a common discussion with dermatologists, the patient finally benefited from an innovative therapy, dupilumab 300 mg every other week after a loading dose of 600 mg subcutaneously. Fastly, the eosinophilic count normalized and pruritus disappeared, whereas KD lesions required more than 1 year of treatment to become undetectable. Dupilumab is still ongoing and effective without notable side effects.

After clear information and with the consent of his relative, the patient gave us his informed consent for publication.

## 3. Discussion

The following literature review was performed on the basis of articles published in English and in French, found using the keywords: “Kimura’s disease” in the PubMed database, from 1988 to 2022.

### 3.1. Clues of KD pathogenesis

Before broaching challenges in diagnosis and treatment, it is necessary to focus on knowledge of KD pathophysiology. Accumulating evidence suggests an immune dysregulation origin characterized by a predominance of T helper 2 (Th2) cytokines both in the blood of patients^[11]^ and directly into lesions.^[12]^ Mast cells and T cells appear to be responsible for cytokines production.^[13]^ Interleukin 5 (IL-5), IL-4, and IL-13 are thought to be involved in the development of KD as they account for 2 biological parameters *i.e.* HE, and high serum IgE levels. IL-5 promotes bone marrow production of eosinophils, prolongs their life span and drives chemotaxis and tissue activation, whereas IL-4 and IL-13 rather mediate isotype switching of B lymphocytes into IgE-producing plasma cells (for review see^[14,15]^). Eosinophils may play a pivotal role in organ disease. Evidence of their activation displayed by immunostaining of eosinophil cationic protein and eosinophil major basic protein in skin or lymph node biopsies is not lacking.^[2,16]^ Furthermore, it has been shown the dosage of granule proteins in the serum acts as a surrogate for KD activity. Increased serum levels of eosinophilic proteins are correlated with disease flares.^[2,17]^ In addition to their putative role in the immune orchestration leading to KD, mast cells, through their IgE-mediated activation, could also be directly implicated in clinical manifestations, such as itching skin rash.^[9]^ To our knowledge, tryptase blood levels have not yet been studied in KD flares.

The triggers responsible for the immunological dysregulation linked to KD remain elusive. The presence of ectopic germinal centers points to an antigen-driven process. The localization of pseudotumors in the salivary glands and head skin, directly at the interface with the external environment, suggests extrinsic antigen involvement. The absence of other autoimmune diseases described in patients affected by KD and no detection of autoimmune markers such as antinuclear antibodies, discredit the hypothesis of a self-antigen driving component in KD.

Biological features of KD may indicate a hypersensitivity reaction to an unknown allergen, more precisely, a type 1 reaction because of infiltrating mast cells and IgE reticulation in lesions.^[13]^ Interestingly, atopy-related diseases, such as allergic rhinitis, allergic asthma, and atopic dermatitis were found in one-third of KD patients, suggesting a similar pathological process. In addition, exacerbation of atopic manifestations occurring in the meantime of KD swelling flares, has been described in some patients. Putative latent infectious agents have been suggested as triggers, such as parasites, *Candida albicans*, Epstein–Barr virus, Human Herpes virus 8 and more recently, based on high-throughput DNA sequencing, Human Polyomavirus-6.^[18,19]^ However, published data remain controversial, and no clear pathogenic relationship has been yet established. It would be interesting to precise whether chronic infections influence KD development, or whether they are concomitant opportunistic agents. In the first situation, anti-microbial therapeutics could be assessed if KD is a reactive process. Currently, we cannot rule out that KD is a subtype of lymphoproliferative disorders characterized by T-cell acquired intrinsic defects, potentially promoted by initial exposure to a foreign antigen.

### 3.2. Insights into the diagnostic challenge

Clinicians and pathologists, particularly in Western countries, are unaware of KD. Considering KD manifestations, the first hypothesis evoked is often a malignant etiology based on an impressive clinical presentation.

Imaging features have been studied to make a preoperative diagnosis. KD lesions appear ill-defined lesions without necrosis or calcification. Various degrees of enhancement depend on the vascular or fibrosis components.^[20]^ Unfortunately, no specific density or signal pattern can discriminate KD on computed tomography scan or MRI. In contrast, MRI findings such as subcutaneous invasion and inhomogeneities of salivary glands, suggesting a malignant process, are frequently found in KD^[21]^ (Fig. 2).

Histological analyses remain central in making the diagnosis of KD, but they are not pathognomonic. A large inflammatory cellular infiltrate, vascular proliferation of the postcapillary venules, and a variable fibrocollagenous component can be found in mimickers. To overcome these difficulties, a generous specimen is required for initial diagnosis. The use of fine-needle aspiration should be limited to recurrence once the diagnosis of KD is well-established.^[22]^

Overall, none of the previously mentioned histological, paraclinical and clinical features are completely specific to KD. The diagnosis should be retained after exclusion of mimickers. A non-exhaustive list of differential diagnoses includes carcinoma, metastasis of solid cancer, lymphoma, multicentric Castleman’s disease, and benign conditions such as hemangioma, histiocytosis, angiolymphoid hyperplasia with eosinophilia and IgG4 related-disease. In this study, we chose not to speak about angiolymphoid hyperplasia with eosinophilia and IgG4 related-disease, broadly addressed in the literature, in favor of hematological malignancies, which represent challenging diagnoses to rule out.

Missing a lymphoma can lead to severe consequences, whereas its overdiagnosis may lead to unwarranted chemotherapy. KD sometimes presents as isolated lymphadenopathy.^[23]^ As KD lesions affect the neck-head region of young people, the disease mimics the clinical presentation of indolent mucosa-associated lymphoid tissue lymphoma and Hodgkin’s lymphoma. Positron emission tomography scan imaging cannot help to distinguish KD from a lymphoma because KD has a significant 18-fluorodeoxyglucose uptake with a reported standardized uptake value max between 4 and 12.^[24]^ Histologically, some lymphoma subtypes, such as Hodgkin’s lymphoma and angioimmunoblastic T-cell lymphoma (AITL), could be difficult to distinguish from KD. The pitfall resides in the heavily reactive microenvironment, in which as little as 1% of malignant cells are lost. Furthermore, AITL was initially misclassified as a reactive disease and molecular clonality assays help us to establish the neoplastic nature of AITL. Can the frontier between KD and lymphoproliferative disorders be reassessed? Only 1 isolated study found a clonal delta T-cell receptor gene rearrangement in a lymph node sample but not in the peripheral blood of a patient affected by KD.^[25]^ Three other publications showed a polyclonal molecular pattern of T-cells in favor of the reactive component.^[26–28]^ Overall, over the past 20 years, the clonality question has been addressed in only 7 patients. Currently, KD is considered as a reactive process, however we believe that KD could be an authentic T-cell lymphoproliferative disorder. To address this issue, it is crucial to analyze clonality in larger patient series and to perform next-generation-sequencing using lymphoid panels.

**Figure 3.**
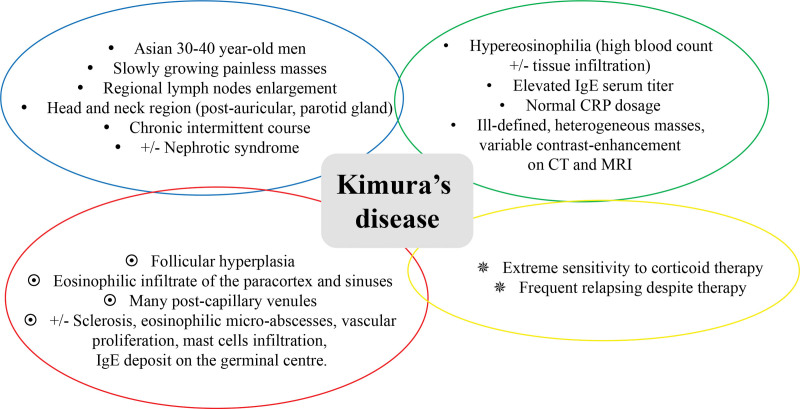
Diagnostic process of KD. A combination of clinical and epidemiological data (blue circle), biological and radiological analyses (green circle), main histological features (red circle), and therapeutic response (yellow circle) were used to make an accurate diagnosis. KD = Kimura's disease.

Even if no consensual diagnostic criteria exists, the diagnostic process must combine strict histological features, clinical information and biological tests (Fig. 3). Although pivotal, pathological findings are insufficient to establish a diagnosis of KD. Pathologists should discuss the patient’s clinical and biological features with clinicians to increase the likelihood of accurate diagnosis. An important step is the rigorous exclusion of diseases that exhibit clinicopathological characteristics mimicking KD. Diagnosing KD is still a great achievement, as highlighted by the long diagnosis time in the above case. Interestingly, the oldest patients have the longest time to diagnosis.^[29]^ For patients aged > 40 years, the diagnostic delay is approximatively 7 years. An increase in the incidence of mimickers and a more atypical KD presentation in the older population may account for this delay.

### 3.3. Therapeutic modalities

Data concerning KD treatments come from the analysis of individual management and, at best, short retrospective case series, exposing inherent publication bias. Standardized response criteria are lacking for comparison studies.

As KD frequently exhibits an indolent chronic course, therapeutic modalities should not be toxic while preserving quality of life. KD affects the head and neck region, therefore, treatments are often required for cosmetic concerns, even though real disfiguring tumors are described in young adults.^[30]^ A particular situation of renal involvement should be distinguished from a localized disease because systemic treatments are absolutely justified in this situation.

Backbone treatment for KD relies on 3 well-documented options: surgical excision, radiotherapy, and systemic glucocorticoids.

Surgery is the first-line treatment, because it is used for both diagnostic and therapeutic purposes. To be eligible for curative surgery, the tumor must be unique and non-infiltrative. KD lesions are often ill-defined, therefore, negative borders are difficult to obtain. A monocentric cases study reported 37.5% relapse after surgery alone with negative surgical margins, and Zhang et al^[31]^ observed nearly 46% recurrence in 13 Chinese patients treated in the same condition. In a French KD cohort, surgical excision without precision on boundary, exhibited 100% response rate, but 60% relapse.^[23]^ Although positive margins could favor relapse after surgery, no study has clearly demonstrated their negative impact on KD evolution.

Radiation has been particularly applied for treating local KD recurrences.^[32]^ Several authors have agreed to propose total doses ranging from 26 to 30 Gy,^[33]^ limited to a maximum of 45 Gy with a conventional fractionation scheme. Weak radiation of approximately 12 Gy was ineffective. No study has reported radiation-induced carcinogenesis, but follow-up often remains short in case series. KD is highly radiosensitive, as shown by 90% response rate to 30 Gy radiation in a retrospective study of 20 patients.^[33]^ In addition to powerful lesion shrinkage, radiation treatment seems to significantly decrease the recurrence rate. Kim et al^[34]^ found 11% recurrence rate in relapsing patients treated with radiotherapy compared to 75% recurrence in those receiving steroids alone, with follow ups from 2 to 9 years. Long-duration control is usually achieved, sometimes for more than 10 years. Furthermore, Ye et al published a meta-analysis showing that surgery followed by post-operative low-dose radiotherapy significantly reduced local recurrences compared to each treatment alone. Radiotherapy is an efficient treatment for salvage conditions by boosting and extending surgery and steroid effect.^[31,33]^ From our perspective, moderate radiation doses should not be excluded because of the benign nature of the KD.

A non-interventional “watch and wait” management may be appropriate for patients with a stable disease course without any systemic condition. Asymptomatic lymphadenopathy or mild cutaneous swelling does not require systematic therapy. Due to the unpredictable development of renal damage associated with KD, a regular renal function and proteinuria assessments must be performed.

Corticosteroid therapy is remarkably effective in KD patients. Glucocorticoids have been widely used in patients with systemic conditions including multifocal lesions and renal damage occurrence.^[35]^ Kottler et al^[23]^ reported 100% response in 13 patients treated with oral corticosteroids as monotherapy. Almost half of the patients experienced complete benefit, while the remaining patients showed partial improvement in clinical tumorous manifestations. The response time is fast, and a steroid effect should be observed during the first 2 weeks of treatment. Precise treatment modalities have not yet been standardized. Oral glucocorticoids starting at a daily dose of 0.5 to 1 mg/kg may be maintained during the first month, after which a tapering regimen should be performed for a personalized total medication period, often 3 to 6 months. For KD-associated nephrotic syndrome, doses higher than 2 mg/kg/d do not seem to be necessary, but some authors agreed to propose extended treatment for nearly over 1 year.^[36,37]^ Relapse usually persists after discontinuation of corticosteroid courses, displaying a suspensive action. Depending on studies, 50% to 74% recurrences were reported with variable delays. Retreatment with steroids is feasible because lesions maintain their cortico-sensitivity.^[38]^ Consequently, some clinicians obtained a disease control thanks to a steroid maintenance regimen of 5 to 10 mg/d associated with short-duration high-dose steroids during exacerbations. For example, Komine and Tamaki^[16]^ reported the use of a successful daily dose of 5 mg prednisolone for up to 6 years.

“True” glucocorticoids resistance is uncommon in KD whereas glucocorticoids dependency remains frequent. Efforts have been made to identify steroid-sparing agents for the management of disease relapse. Three categories of molecules have been tested: anti-allergy drugs, immunomodulatory agents, and more recently targeted therapies (Table 1).

**Table 1:** Alternative systemic therapeutic options to corticoids in KD.

Drug	Duration	Associated-drug	Clinical response*	Biological response†	Delay for response	Recurrence post treatment and follow-up duration	Main lesion	Renal damage	Side effect	Patient age and gender	Timing of drug use	References
Cetirizine 10 mg/d	–	Azathioprine?	Complete	Normal eosinophil count	Two months	No recurrence 6 mo	Arm and neck tumors	–	None	42-year-old woman	Fourth line	Ben-Chetrit et al^[39]^
Cetirizine 10 mg/d	20 mo	None	Complete and partial depending on the lesion	–	–	–	Thigh masses	30 years ago	None	45-year-old man	First line	Kumar et al^[40]^
Mycophenolate mofetil 2000 mg/d then 1000 mg/d	23 mo	Topical timolol solution	Partial	Partial	One month	–	Head region masses	–	Neutropenia	32-year-old man	First line	Shah et al^[41]^
Ciclosporine 5 mg/kg/d then gradually reduced	6 mo	None	Complete	Complete	One week	No recurrence 18 mo	Neck and earlobes masses	–	None	29-year-old woman	Third line	Kaneko et al^[42]^
Ciclosporine 2.5 mg/kg/d for 1 wk then 5 mg/kg/d for 2 wk	3 wk	None	Complete	Partial	One week	Recurrence for some lesions	Neck, bilateral elbows and groin masses	–	None	40-year-old female	Fourth line	Wang et al^[43]^
Mepolizumab 300 mg/mo	8 mo	–	Partial	Partial	–	–	Upper limb masses	–	None	42-year-old woman	Third line	Kinoshita et al^[44]^
Dupilumab 600 mg loading dose then 300 mg/wk then 300 mg/mo	14 mo	–	Near complete	Complete	Four months to achieve complete response	–	Single forearm masse	–	None	57-year-old man	First line	Teraki et al^[45]^

KD = Kimura's disease.

* Clinical response was classified as complete when the article mentioned the total disappearance of KD physical signs (mainly tumoral syndrome) and as partial when there was at least 50% of decrease.

† A complete biological response corresponds to the normalization of eosinophil and IgE levels. A partial biological response indicated a decrease in eosinophil and IgE levels.

Based on the likely allergic origin, treatments that inhibit mast cells mediators have been used. Cetirizine, a selective histamine H1-receptor blocker, provided promising findings in 1 study, as it allowed the discontinuation of corticoids without recurrences for 6 months, possibly by modulating inflammation and eosinophil influx.^[39]^ However, others case reports and our experience showed that cetirizine alone did not work efficiently in diminishing the masses.^[40]^ The quality of life was greatly improved by relieving itching, and tolerance was excellent. Omalizumab, an anti-IgE antibody, has been used in relapsing KD, however, after several months of monotherapy, MRI controls showed a modest decrease in masses.^[46]^ Taken together, anti-allergic drugs may be an additional treatment to control allergic symptoms even without an atopic background, but they do not seem to reduce the size of tumors.

Immunosuppressive agents work by suppressing lymphocyte activation, and by the same way the exuberant Th2 cytokines-mediated immune response. Antimetabolite drugs can work alone, as shown for mycophenolate mofetil^[41]^ but expose patients to neutropenia. Scattered case reports favorably evaluated azathioprine^[39,47]^ and methotrexate^[48]^ in KD. Cyclophosphamide, known for its immunosuppressive properties, was administered with glucocorticoids in severe case of kidney failure and, most of the time, worked.^[49]^ The risk of induced leukemogenesis limits its long-term use. The majority of the available data concerns ciclosporine in patients with multiple relapsing tumors. Prolonged successful responses were observed but recurrences were still possible in nearly one-third of the patients.^[23,42,43,50,51]^ Other reports employed ciclosporine in severe forms of KD with renal involvement.^[47,52]^ The doses used were quite high, and many studies did not report monitoring of residual concentration except one suggesting that 75 ng/ml was sufficient for therapeutic response.^[50]^ A long list of serious side effects is known, ranging from frequent kidney injury to seldom thrombotic microangiopathy. In return, one important factor is how quickly the first signs of clinical response were obtained. Shrinkage of masses after less than 1 week of treatment suggested that ciclosporine could be useful in urgent situations. Evidence of selectively and dose-dependent suppression of Th2 cytokine overexpression by ciclosporine provides a pathophysiological rationale for this therapy.^[50]^ Overall, these drugs were always used concomitantly with other treatments, making it difficult to clearly determine their effectiveness. According to us, these immunosuppressive therapies may be suitable in case of severe systemic involvement that are insufficiently controlled by steroids. In the latter condition, and when patients develop contraindications to glucocorticoids, ciclosporine appears to be a good option. These findings support a strong T cell-driven role in KD pathogenesis. Of note, B cells seem to play a lesser role, as demonstrated by the nearly no effect of rituximab on KD tumors despite B cell depletion.^[53]^

The marked eosinophilia observed in KD is not a simple bystander phenomenon but an essential part of KD pathogenesis. As IL-5 is a critical regulator of eosinophilic survival, proliferation, and function, its inhibition may be a logical therapeutic target for KD. In hypereosinophilia syndrome, mepolizumab, an anti–interleukin-5 monoclonal antibody, showed efficacy as a corticosteroid-sparing drug.^[54]^ The first report of successful treatment with mepolizumab was in a patient with KD and an ulcerative colitis.^[5]^ Recently, 1 study corroborated the efficacy of mepolizumab on eosinophils blood count and tissue infiltration, but without reduction of masses swelling.^[44]^ Used as a fourth line therapy, benralizumab, an anti-IL5 receptor monoclonal antibody responsible for clearance of eosinophils through antibody-dependent cell cytotoxicity, prevented the recurrence of KD in a patient with severe asthma and atopic dermatitis.^[55]^ Interestingly, most of these studies presented atypical KD with unusually affected organs or severe allergic symptoms justifying the use of anti-eosinophil therapy. To go further, it would be interesting to test mepolizumab in front line for typical KD and to act specifically on the “eosinophils/Th2 cells axis” which is presumed to be the core of KD pathogenesis. A combination of anti-IL-5 drugs with immunosuppressants such as ciclosporine could be tried. With a safe tolerance profile, dupilumab, an antibody blocking the IL-4 receptor alpha chain, has been successfully assessed for KD treatment.^[45,56,57]^ The high efficiency of dupilumab in KD suggests that the global Th2 inflammation pathway is critical for KD pathogenesis, and not only for eosinophilic final activation.

**Figure 4.**
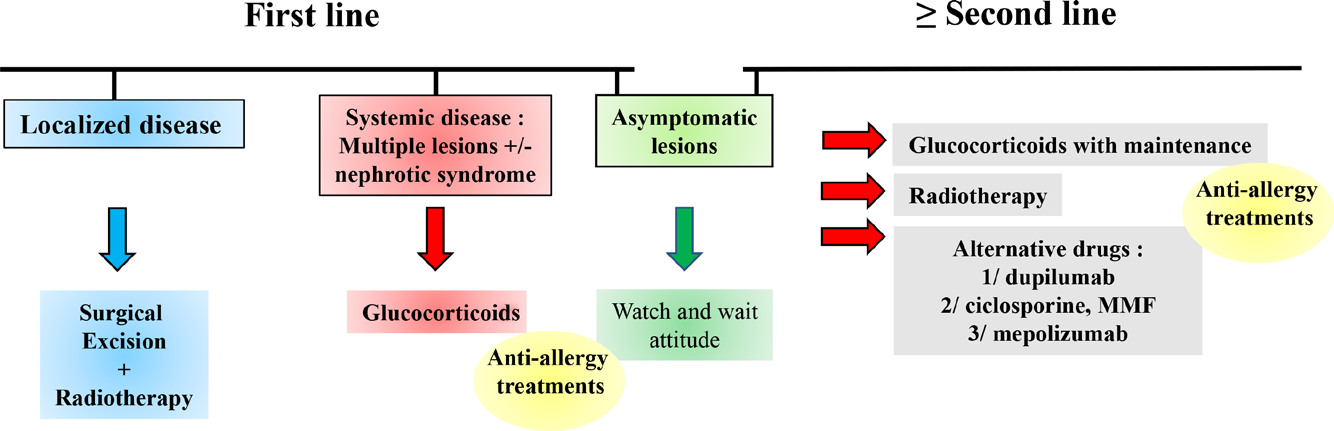
A proposed therapeutical plan to manage KD. The first-line treatment may consist of surgical excision with adjuvant low-dose radiotherapy if the KD lesion is unique, whereas multiple lesions and or systemic KD, mainly represented by nephrotic syndrome, must be treated with glucocorticoids. Several options are available for relapsing KD, such as long-term glucocorticoid therapy, radiotherapy when possible, and alternative drugs alone or in combination with glucocorticoids. Asymptomatic and stable lesions are eligible for monitoring. Kimura's disease, MMF = mycophenolate mofetil.

To conclude, there are no standardized treatment guidelines because of the seldom condition of KD restricting the use of large-scale prospective randomized controlled trials. Therefore, we only report and discuss retrospective data of clinical cases. Case-control or at best prospective studies would be required to clearly compare therapeutic options in KD. Considering these limitations, we present a treatment protocol in Figure 4. Well-known therapeutic options include surgical excision, systemic glucocorticoids, and radiotherapy alone or in combination. Among them, the association of glucocorticoids and low-dose radiotherapy has been pointed out by observational case series studies as the most effective first-line treatment for non-systemic KD. This combination resulted in a prolonged disease-free survival period. When this modality cannot be realized, in cases of multiple masses or nephrotic syndrome, glucocorticoids are useful in first line treatment. The main challenge for clinicians is managing disease relapse. Regardless of therapy received, an high recurrence rate, from 25% to often up to 50%, is observed, depending on case series. From the available data, it seems that a specific patient group could be individualized on the basis of multiple recurrences, whereas other patients tended to be cured in 1 shot. Our patient belongs to the first group, with more than 6 recurrences over 20 years. Finding predictive factors to identify such patients may provide guidance for therapeutic management. Patients in the relapse group should benefit from initial high curative therapy. Although nonspecific, glucocorticoids are still the most powerful anti-inflammatory molecules to manage KD flares.^[58]^ Maintenance protocols may prevent relapse during cessation of treatment. Promising advances have emerged from the hypereosinophilia and the allergic fields, notably with the great effect of dupilumab. Long-term follow-up and evaluation in several patients with KD will be required to confirm the high and sustained efficacy of dupilumab. Deciphering the physiopathology of KD by studying other key cells, such as mast cells, will help to find new therapies.

## Author contributions

**Conceptualization:** Stephanie Cordeil, Arnaud Hot.

**Methodology:** Arnaud Hot.

**Supervision:** Olivier Hermine.

**Validation:** Olivier Hermine, Arnaud Hot.

**Writing – original draft:** Stephanie Cordeil.

**Writing – review & editing:** Stephanie Cordeil, Olivier Hermine, Arnaud Hot.

## References

[1] Valent P. Pathogenesis, classification, and therapy of eosinophilia and eosinophil disorders. Blood Rev. 2009;23:157–65. 19246139 10.1016/j.blre.2009.01.001

[2] Ohta N Okazaki S Fukase S. Serum concentrations of eosinophil cationic protein and eosinophils of patients with Kimura’s disease. Allergol Int. 2007;56:45–9. 17259809 10.2332/allergolint.O-06-442

[3] Barge S Bouchachi A Bechara C. Eosinophilia with stroke in a Chinese patient: Kimura’s disease complicated with fibroblastic endocarditis, first report. Am J Hematol. 2008;83:432. 18418903 10.1002/ajh.21121

[4] Nakagawa C Sakaguchi Y Nakajima T. A case of eosinophilic myocarditis complicated by Kimura’s disease (Eosinophilic Hyperplastic Lymphogranuloma) and Erythroderma. Jpn Circ J. 1999;63:141–4. 10084379 10.1253/jcj.63.141

[5] Al Shammari F Nasiri A Alkhathami M. Mepolizumab as an effective treatment for Kimura’s disease associated with ulcerative colitis: a case report. J Family Med Prim Care. 2019;8:3028–31. 31681687 10.4103/jfmpc.jfmpc_373_19 PMC6820387

[6] Shimamoto C Takao Y Hirata I. Kimura’s disease (angiolymphoid hyperplasia with eosinophilia) associated with ulcerative colitis. Gastroenterol Jpn. 1993;28:298–303. 8486218 10.1007/BF02779234

[7] Whelan TV Maher JF Kragel P. Nephrotic syndrome associated with Kimura’s disease. Am J Kidney Dis. 1988;11:353–6. 3354572 10.1016/s0272-6386(88)80143-4

[8] Chen Y Wang J Xu F. Clinicopathological features and prognosis of Kimura’s disease with renal involvement in Chinese patients. Clin Nephrol. 2016;85:332–9. 27142199 10.5414/CN108749

[9] Hui PK Chan JK Ng CS. Lymphadenopathy of Kimura’s disease. Am J Surg Pathol. 1989;13:177–86. 2919716 10.1097/00000478-198903000-00001

[10] Chen H Thompson LDR Aguilera NSI. Kimura disease: a clinicopathologic study of 21 cases. Am J Surg Pathol. 2004;28:505–13. 15087670 10.1097/00000478-200404000-00010

[11] Yamazaki K Kawashima H Sato S. Increased CD45RO+ CD62L+ CD4+ T-cell subpopulation responsible for Th2 response in Kimura’s disease. Hum Immunol. 2013;74:1097–102. 23747992 10.1016/j.humimm.2013.06.001

[12] Ohta N Fukase S Suzuki Y. Increase of Th2 and Tc1 cells in patients with Kimura’s disease. Auris Nasus Larynx. 2011;38:77–82. 20554415 10.1016/j.anl.2010.03.011

[13] Kimura Y Pawankar R Aoki M. Mast cells and T cells in Kimura’s disease express increased levels of interleukin-4, interleukin-5, eotaxin and RANTES. Clin Exp Allergy. 2002;32:1787–93. 12653173 10.1046/j.1365-2222.2002.01552.x

[14] Valent P Gleich GJ Reiter A. Pathogenesis and classification of eosinophil disorders: a review of recent developments in the field. Expert Rev Hematol. 2012;5:157–76. 22475285 10.1586/ehm.11.81 PMC3625626

[15] McCormick SM Heller NM. Commentary: IL-4 and IL-13 receptors and signaling. Cytokine. 2015;75:38–50. 26187331 10.1016/j.cyto.2015.05.023 PMC4546937

[16] Komine M Tamaki K. Kimura’s disease with prolonged history and prominent vascular involvement. Acta Derm Venereol. 2005;1:1–1. 10.1080/00015550510027414 16191866

[17] Morita H Kitano Y. Kimura’s disease with high serum levels of eosinophil cationic protein and major basic protein. Clin Immunol Immunopathol. 1994;72:280–1. 8050202 10.1006/clin.1994.1142

[18] Kemény E Nagy SZ Nagy F. Membranous nephropathy accompanied by HHV8-DNA-positive angiolymphoid hyperplasia of the skin with eosinophilia: lack of HHV8 viral DNA in the kidney biopsy. Clin Nephrol. 2004;61:295–6. 15125037 10.5414/cnp61295

[19] Rascovan N Bouchard S Grob JJ. Human polyomavirus-6 infecting lymph nodes of a patient with an angiolymphoid hyperplasia with eosinophilia or Kimura disease. Clin Infect Dis. 2016;62:ciw135. 10.1093/cid/ciw135 26962076

[20] Zhang R Ban XH Mo YX. Kimura’s disease: the CT and MRI characteristics in fifteen cases. Eur J Radiol. 2011;80:489–97. 21030173 10.1016/j.ejrad.2010.09.016

[21] Iwai H Nakae K Ikeda K. Kimura disease: diagnosis and prognostic factors. Otolaryngol Head Neck Surg. 2007;137:306–11. 17666261 10.1016/j.otohns.2007.03.027

[22] Chow LT Yuen RW Tsui WM. Cytologic features of Kimura’s disease in fine-needle aspirates. A study of eight cases. Am J Clin Pathol. 1994;102:316–21. 8085555 10.1093/ajcp/102.3.316

[23] Kottler D Barète S Quéreux G. Retrospective multicentric study of 25 kimura disease patients: emphasis on therapeutics and shared features with cutaneous IgG4-related disease. Dermatology. 2015;231:367–77. 26452023 10.1159/000439346

[24] Yang TH Chou YH Kao WY. Kimura disease simulating Hodgkin’s lymphoma on 18F FDG PET-CT: report of a case. Nucl Med Mol Imag. 2014;48:313–6. 10.1007/s13139-014-0285-1 PMC4571665 26396638

[25] Chim CS Fung A Shek TWH. Analysis of Clonality in Kimura’s disease. Am J Surg Pathol. 2002;26:1083–6. 12170098 10.1097/00000478-200208000-00016

[26] Jang KA Ahn SJ Choi JH. Polymerase chain reaction (PCR) for human herpesvirus 8 and heteroduplex PCR for clonality assessment in angiolymphoid hyperplasia with eosinophilia and Kimura’s disease. J Cutan Pathol. 2001;28:363–7. 11437942 10.1034/j.1600-0560.2001.280705.x

[27] Chim CS Shek W Liang R. Kimura’s disease: no evidence of clonality. Br J Ophthalmol. 1999;83:880–1. 10636669 10.1136/bjo.83.7.878c PMC1723120

[28] Chim CS Liang R Fung A. Further analysis of clonality in Kimuraʼs disease. Am J Surg Pathol. 2003;27:703–4. 12717259 10.1097/00000478-200305000-00018

[29] Kakehi E Kotani K Otsuka Y. Kimura’s disease: effects of age on clinical presentation. QJM. 2020;113:336–45. 31800058 10.1093/qjmed/hcz312

[30] Kapoor NS O’Neill JP Katabi N. Kimura disease: diagnostic challenges and clinical management. Am J Otolaryngol. 2012;33:259–62. 21763034 10.1016/j.amjoto.2011.05.005

[31] Zhang G Li X Sun G. Clinical analysis of Kimura’s disease in 24 cases from China. BMC Surg. 2020;20:1. 31898499 10.1186/s12893-019-0673-7 PMC6941305

[32] Itami J Arimizu N Miyoshi T. Radiation therapy in Kimura’s disease. Acta Oncol. 1989;28:511–4. 2789827 10.3109/02841868909092260

[33] Hareyama M Oouchi A Nagakura H. Radiotherapy for Kimura’s disease: the optimum dosage. Int J Radiat Oncol Biol Phys. 1998;40:647–51. 9486615 10.1016/s0360-3016(97)00813-4

[34] Kim GE Kim WC Yang WI. Radiation treatment in patients with recurrent Kimura’s disease. Int J Radiat Oncol Biol Phys. 1997;38:607–12. 9231686 10.1016/s0360-3016(97)89487-4

[35] Ren S Li XY Wang F. Nephrotic syndrome associated with Kimura’s disease: a case report and literature review. BMC Nephrol. 2018;19:316. 30409112 10.1186/s12882-018-1123-y PMC6225567

[36] Matsuda O Makiguchi K Ishibashi K. Long-term effects of steroid treatment on nephrotic syndrome associated with Kimura’s disease and a review of the literature. Clin Nephrol. 1992;37:119–23. 1563115

[37] Shehwaro N Langlois AL Gueutin V. La maladie de Kimura: une cause méconnue de syndrome néphrotique à lésions glomérulaires minimes de l’adulte. Néphrol Thérap. 2014;10:46–50. 24360981 10.1016/j.nephro.2013.09.001

[38] Nakahara C Wada T Kusakari J. Steroid-sensitive nephrotic syndrome associated with Kimura disease. Pediatr Nephrol. 2000;14:482–5. 10872189 10.1007/s004670050798

[39] Ben-Chetrit E Amir G Shalit M. Cetirizine: an effective agent in Kimura’s disease. Arthritis Rheum. 2005;53:117–8. 15696573 10.1002/art.20908

[40] Kumar V Mittal N Huang Y. A case series of Kimura’s disease: a diagnostic challenge. Ther Adv Hematol. 2018;9:207–11. 30013767 10.1177/2040620718780370 PMC6041861

[41] Shah K Tran AN Magro CM. Treatment of Kimura disease with mycophenolate mofetil monotherapy. JAAD Case Rep. 2017;3:416–9. 28932783 10.1016/j.jdcr.2017.04.010 PMC5594227

[42] Kaneko K Aoki M Hattori S. Successful treatment of Kimura’s disease with cyclosporine. J Am Acad Dermatol. 1999;41(5 Pt 2):893–4. 10534681 10.1016/s0190-9622(99)70354-3

[43] Wang YS Tay YK Tan E. Treatment of Kimura’s disease with cyclosporine. J Dermatolog Treat. 2005;16:242–4. 16249147 10.1080/09546630510044003

[44] Kinoshita M Ogawa Y Onaka M. Mepolizumab-responsive Kimura disease. J Allergy Clin Immunol Pract. 2021;9:2928–30. 33684634 10.1016/j.jaip.2021.02.049

[45] Teraki Y Terao A. Treatment of Kimura disease with dupilumab. JAMA Dermatol. 2022;158:329–30. 35138349 10.1001/jamadermatol.2021.5885

[46] Nonaka M Sakitani E Yoshihara T. Anti-IgE therapy to Kimura’s disease: a pilot study. Auris Nasus Larynx. 2014;41:384–8. 24406057 10.1016/j.anl.2013.12.006

[47] Senel MF Van Buren CT Etheridge WB. Effects of cyclosporine, azathioprine and prednisone on Kimura’s disease and focal segmental glomerulosclerosis in renal transplant patients. Clin Nephrol. 1996;45:18–21. 8616952

[48] Ma H. Treatment of Kimura’s disease with oral corticosteroid and methotrexate. An Bras Dermatol. 2020;95:115–7. 31889595 10.1016/j.abd.2019.03.006 PMC7058860

[49] Wang DY Mao JH Zhang Y. Kimura disease: a case report and review of the Chinese literature. Nephron Clin Pract. 2009;111:c55–61. 19052471 10.1159/000178980

[50] Katagiri K Itami S Hatano Y. In vivo expression of IL-4, IL-5, IL-13 and IFN-gamma mRNAs in peripheral blood mononuclear cells and effect of cyclosporin A in a patient with Kimura’s disease. Br J Dermatol. 1997;137:972–7. 9470918

[51] Dai L Wei XN Zheng DH. Effective treatment of Kimura’s disease with leflunomide in combination with glucocorticoids. Clin Rheumatol. 2011;30:859–65. 21286771 10.1007/s10067-011-1689-2

[52] PubMed entry. Available at: http://www.ncbi.nlm.nih.gov/pubmed/10872189 [access date August 13, 2020].

[53] Ghosn S Bahhady R Mahfouz R. Concomitant occurrence of Kimura disease and mycosis Fungoides in a Lebanese woman: significance and response to rituximab. Am J Dermatopathol. 2009;31:814–8. 19786854 10.1097/DAD.0b013e3181acedf8

[54] Rothenberg ME Klion AD Roufosse FE.; Mepolizumab HES Study Group. Treatment of patients with the hypereosinophilic syndrome with mepolizumab. N Engl J Med. 2008;358:1215–28. 18344568 10.1056/NEJMoa070812

[55] Szeto VG Chin-Yee B Dehghani M. Successful treatment of Kimura disease with benralizumab. Ann Hematol. 2022;101:2099–100. 35622096 10.1007/s00277-022-04873-0

[56] Huang HY Yang CY Yao WT. Kimura disease of the thigh treated with surgical excision and dupilumab. Ann Plast Surg. 2022;88:S110–3. 35225857 10.1097/SAP.0000000000003106

[57] Bellinato F Mastrosimini MG Querzoli G. Dupilumab for recalcitrant Kimura disease. Dermatol Ther. 2022;35:e15674. 35770515 10.1111/dth.15674

[58] Zen M Canova M Campana C. The kaleidoscope of glucorticoid effects on immune system. Autoimmun Rev. 2011;10:305–10. 21224015 10.1016/j.autrev.2010.11.009

